# Novel copy number variation of *COLQ* gene in a Moroccan patient with congenital myasthenic syndrome: a case report and review of the literature

**DOI:** 10.1186/s12883-022-02822-y

**Published:** 2022-08-05

**Authors:** Youssef El Kadiri, Ilham Ratbi, Abdelaziz Sefiani, Jaber Lyahyai

**Affiliations:** 1 Research Team in Genomics and Molecular Epidemiology of Genetic Diseases, Genomics Center of Human Pathologies, Faculty of Medicine and Pharmacy, Mohammed V University in Rabat, 10 100 Rabat, Morocco; 2 Department of Medical Genetics, National Institute of Health, BP 769-Agdal, 10 090 Rabat, Morocco

**Keywords:** Congenital myasthenic syndrome, *COLQ* gene, Novel CNV, Protein-protein interaction, Case report

## Abstract

**Background:**

Congenital myasthenic syndromes (CMSs) are rare genetic diseases due to abnormalities of the neuromuscular junction leading to permanent or transient muscle fatigability and weakness. To date, 32 genes were found to be involved in CMSs with autosomal dominant and/or recessive inheritance patterns. CMS with acetylcholinesterase deficiency, in particular, was determined to be due to biallelic mutations of *COLQ* gene with early-onset clinical signs. Here, we report clinical features and novel molecular findings of *COLQ*-related CMS in a Moroccan patient with a review of the literature for this rare form.

**Case presentation:**

In this study, we report the case of a 28-month-old Moroccan female patient with hypotonia, associated to axial muscle weakness, global motor delay, bilateral ptosis, unilateral partial visual field deficiency with normal ocular motility, and fatigable muscle weakness. Clinical exome sequencing revealed a novel homozygous deletion of exon 13 in *COLQ* gene, NM_005677.4(COLQ):c.(814+1_815-1)_(954+1_955-1) del p.(Gly272Aspfs*11). This finding was subsequently confirmed by quantitative real-time PCR (qPCR) in the proband and her parents. In silico analysis of protein-protein interaction network by STRING tool revealed that 12 proteins are highly associated to COLQ with an elevated confidence score. Treatment with Salbutamol resulted in clear benefits and recovery.

**Conclusions:**

This clinical observation illustrates the important place of next-generation sequencing in the precise molecular diagnosis of heterogeneous forms of CMS, the appropriate management and targeted treatment, and genetic counseling of families, with a better characterization of the mutational profile of this rare disease in the Moroccan population.

## Background

Congenital myasthenic syndromes (CMSs) are a rare group of clinically and genetically heterogeneous neuromuscular disorders with impaired neuromuscular transmission. Symptoms CMSs usually appear at birth or in early infancy and are characterized by fatigable muscle weakness, fluctuating throughout the day and worsened with exertion [1–4].

To date, 32 CMS-associated genes have been described in the literature with autosomal dominant and/or autosomal recessive transmission. The genetic mutations alter the structure and normal function of 8 presynaptic, 4 synaptic, 15 post-synaptic, and 5 glycosylation proteins located at different motor endplate levels [1].

The gene encoding for the collagen-like tail subunit of asymmetric acetylcholinesterase is among the most common causative CMSs related genes, responsible for AChE deficiency or CMS type-5 (CMS5; MIM #603034 ). It is due to biallelic LoF mutations in the *COLQ* gene (*COLQ*; MIM*603033) causing prolonged synaptic currents and action potentials due to extended residence of acetylcholine in the synaptic space. Treatment with Salbutamol (0.1 mg/kg, in two doses) and Ephedrine (0.5–1 mg/kg/day) are the most effective treatments for *COLQ*-related endplate AChE deficiency, whereas AChE inhibitors such as Pyridostigmine should be avoided due to their inefficiency and the risk of causing worsening of the muscle weakness [5–7].

We present, to the best of our knowledge, the second clinical and molecular genetic diagnosis of CMS type-5 in Morocco and the fourth copy number variation (CNV) worldwide through the case of a Moroccan female patient born to healthy parents (first cousins). She harbored a novel homozygous deletion of exon 13 in the *COLQ* gene identified by the clinical exome sequencing (CES) approach, integrated with a copy number analysis algorithm [8–11]. Four other unrelated CMS patients, previously described in the literature with large exon deletions in the *COLQ* gene are also discussed here.

## Case presentation

**Fig. 1:**
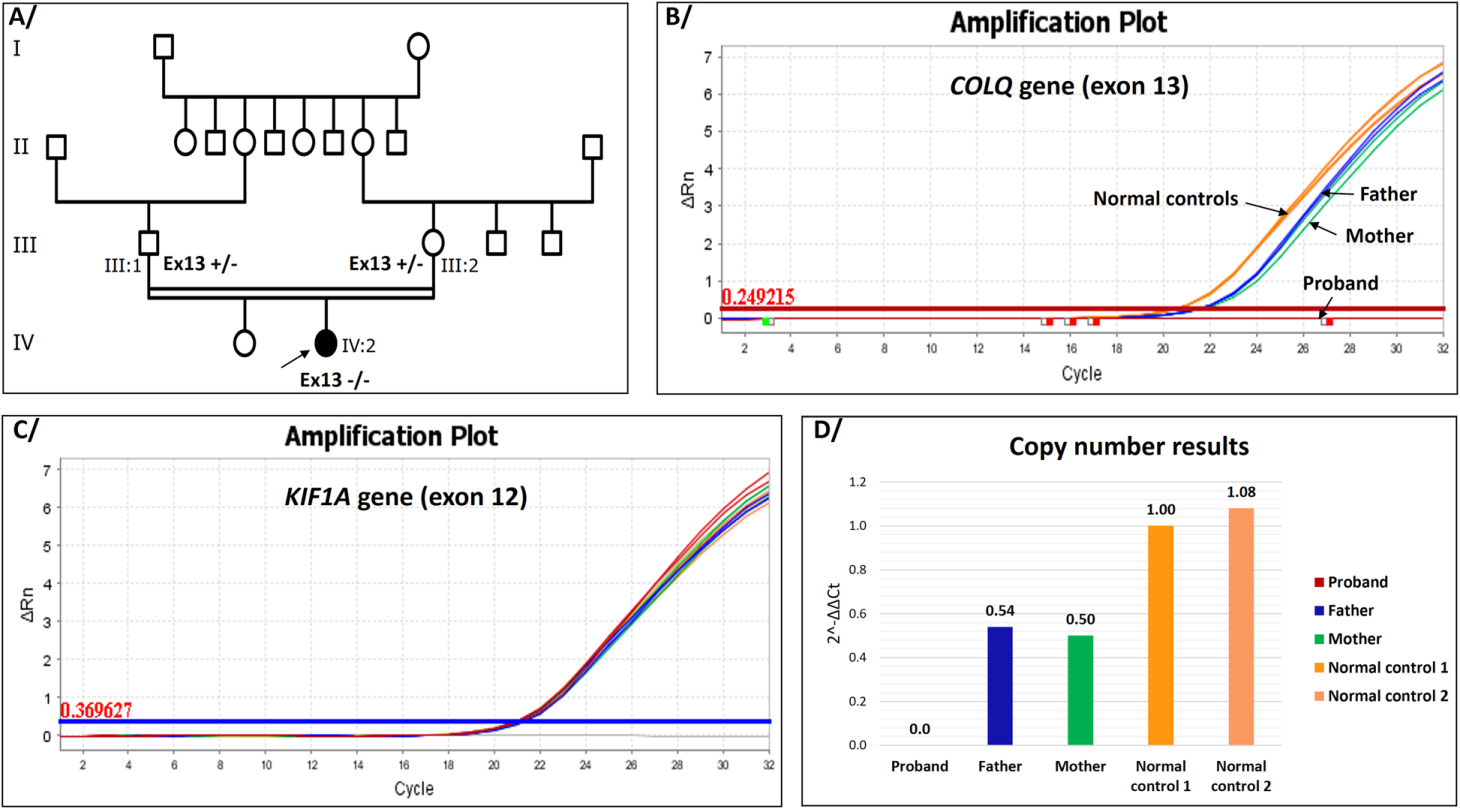
Genealogical and molecular data of the Moroccan family with a novel CNV in the *COLQ* gene. **A** The family pedigree illustrates the affected patient (homozygous for the mutation) and both parents (Heterozygous carriers). The black arrow indicates the proband. **B** The representative amplification plots of the target gene (*COLQ)*. **C** The representative amplification plots of the endogenous gene (housekeeping gene). Both Real-time PCR amplification plots show various amounts of the amplified region in the proband, both parents and two normal controls with determination of mean threshold cycle (Ct) values using QuantStudio^TM^ Real-Time PCR Software v1.7.1 (Applied Biosystems, Thermo Fisher Scientific). **D** Bar graph showed the copy number results calculated from delta-delta Ct

The Moroccan family reported here was referred from pediatrics hospital to our outpatient genetics clinic in Rabat for molecular genetic testing and counseling. The proband (IV:2) was a 28-month-old Moroccan female born to a consanguineous couple, the second child of a healthy couple (III:1, III:2), with no relevant medical history (Fig. 1A). She was born full-term, uneventful spontaneous pregnancy with vaginal delivery. At 2 months of life, she had facial muscle weakness with poor sucking followed by growth retardation (3300g). At 28 months, physical examination showed fatigable muscle weakness with axial hypotonia, bilateral ptosis, unilateral partial visual field deficiency with normal ocular motility, head drop forward, and isolated weakness of the neck extensor muscles. She presented diminished muscular strength and abolished deep tendon reflexes. She had delayed motor developmental milestones with good cognitive development. The electromyography (EMG) showed a significant decremental response on repetitive nerve stimulation testing of deltoid muscle at a frequency of 3 Hz. Laboratory testing was unremarkable for anti-muscle specific kinase (anti-MuSK) and anti-acetylcholine receptor (AChR) antibodies with normal creatine kinase levels in the serum.

CMS diagnosis was considered in the patient (IV:2), but because of the phenotypic and genetic heterogeneity of CMS subtypes, CES was performed as a molecular diagnosis strategy to target causal genes and to exclude the differential diagnoses with congenital myopathy disorders.

Written informed consent was obtained from the proband’s family prior to performing blood sampling and genetic testing.

Next-generation sequencing (NGS) analysis was performed on the genomic DNA of the patient from peripheral venous blood with a clinical exome sequencing panel kit. The Clinical Exome Solution V2 Kit (Sophia Genetics SA, Saint-Sulpice, Switzerland) was used for the enrichment of the conserved coding regions (±5bp of intronic regions) that cover 4490 genes (target region of 12 Mb) related to rare inherited diseases. Paired-end exome sequencing was performed on an Illumina NextSeq® 500 sequencer (San Diego, CA, USA) with a read length of 150 x 2 according to the manufacturer’s protocols. The base calling and image analysis were conducted using Real-Time Analysis software (integrated to the NextSeq 500 system; Illumina). The BCL (base calls) binary is converted into FASTQ utilizing the Illumina package bcl2fastq.

NGS raw data were analyzed using the SOPHiA DDM ^TM^ platform (Sophia Genetics SA) with algorithms for alignment, calling single nucleotide polymorphisms (SNPs) and small insertions/deletions (Pepper^TM^, Sophia Genetics SA patented algorithm), calling copy number variations (Muskat^TM^, Sophia Genetics SA patented algorithm), and annotation. The raw reads were aligned to the human reference genome sequence (GRCh37/hg19), and an integrative genomics viewer (IGV) was used to visualize the binary alignment map (BAM) file.

**Fig. 2:**
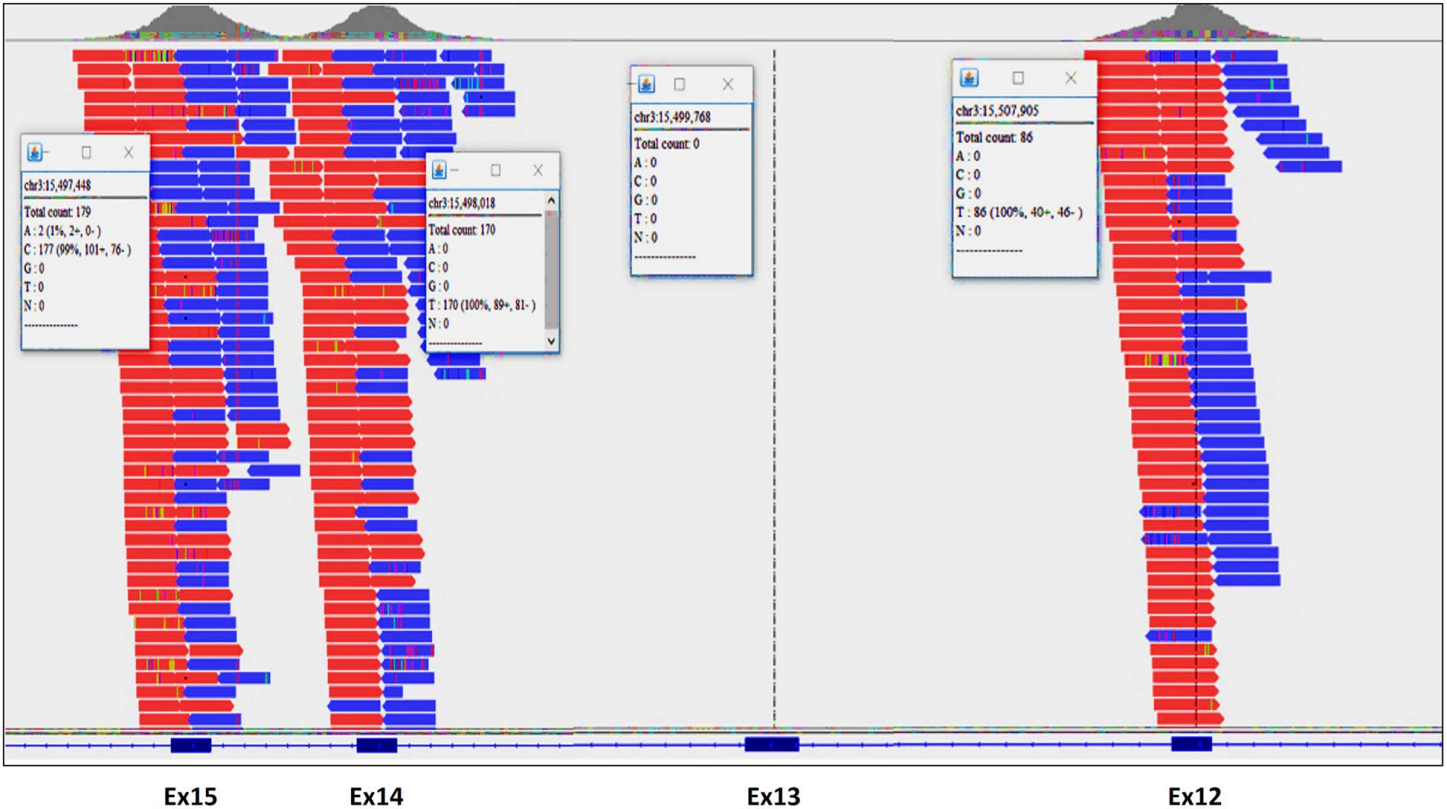
Visualization of the aligned sequencing reads representing deletion of exon 13 in the *COLQ* gene using Integrative Genomics Viewer (IGV) v2.5.0

The bioinformatics analysis of the sequencing of the coding regions by a large panel of 4490 genes revealed the absence of pathogenic or likely pathogenic single nucleotide variants (SNV) in all sequenced genes. In contrast, a novel copy number variation (CNV) was detected in the patient with a high confidence and visualized on IGV (Fig. 2). It corresponds to a deletion of exon 13, NM_005677.4(COLQ):c.(814+1_815-1)_(954+1_955-1) del p.(Gly272Aspfs*11), which results in a frameshift mutation starting from amino acid position 272 and introduces a premature termination signal at codon position 282. The homozygous deletion of exon 13 may lead to the synthesis of a truncated COLQ protein lacking 174 C-terminal amino acid residues if the mRNA escapes from degradation by the nonsense-mediated mRNA decay mechanism (NMD). Otherwise, the truncated protein is rapidly degraded by NMD (post-translational degradation) resulting in both null alleles of *COLQ* gene by LoF mechanism.

The established CNV was cross-checked with the 1000 Genomes Project database (http://www.1000genomes.org/), Exome Variant Server (http://evs.gs.washington.edu/EVS/), ClinVar database (http://www.ncbi. nlm.nih.gov/clinvar/), Human Gene Mutation Database (HGMD) (http://www.hgmd.cf.ac.uk), and LOVD database (https://www.lovd.nl/). It has not been reported in public human databases so far (accessed on 9 May 2022), and it was not found in 138 Moroccan exomes (in-house database).

This copy number change identified in *COLQ* gene was subsequently confirmed by quantitative real-time PCR (qPCR) in the proband and her parents using SYBR® Green dyes and loaded on a QuantStudio^TM^ 7 Flex Real-Time PCR System (Applied Biosystems, Thermo Fisher Scientific). Genomic DNA (gDNA) was extracted through KingFisher ™ Duo Prime System (Thermo Scientific™) using MagMAX™ DNA Multi-Sample Ultra 2.0 Kit (Applied Biosystems™) according to the manufacturer's recommended procedures. gDNA purity and quantity were determined for each sample using NanoDrop™ 2000 Spectrophotometer (Thermo Scientific™), followed by Qubit 3.0 Fluorometer with the Qubit dsDNA HS Assay Kit (Invitrogen, Thermo Fisher Scientific) to accurately measure DNA quantity.

To quantify the region of interest, a pair of primers was designed in such a way that the forward primer (5’-CAAAGGTCGCTCACATCTCC-3’) was hybridized within the intronic sequence (intron12-13) and the reverse primer (5’-GGGCCCATACACAGATTCC-3’) was incorporated within the exon 13 sequence with an amplicon length of 198 base pairs. The reference gene used was *KIF1A* with amplification of exon 12 using the set of primers (ex12_F: 5’-GGAGCAGACATAGCCCTGG-3’ and ex12_R: 5’-CCTAATTCAAGCACGAGAGG-3’).

Each qPCR run included amplification of a normal control with known copy number in the same gene to analyze (exon 13 of *COLQ* gene), and also an amplification of a housekeeping gene (exon 12 of *KIF1A* gene in our study) as copy number control with delta-delta Ct method for copy number calculation. In this case, SYBR Green qPCR (*Powe*r SYBR™ Green PCR Master Mix, Applied Biosystems™) was used to confirm the absence of exon 13 from the affected patient and to quantify it in both parents in order to show that they are heterozygous.

qPCR analysis validated the molecular genetic diagnosis by showing that this novel copy number mutation of exon 13 in *COLQ* gene identified in the proband was inherited from both parents (Fig. 1B-D**)**.

The accession number from ClinVar database, SCV001976653 was assigned to our novel mutation.

After molecular analysis and identification of the causative gene, the proband showed significant clinical improvement following treatment with oral Salbutamol. This drug, a Selective Beta 2-Adrenergic Receptor Agonist, discloses positive effects on muscle strength. Currently, after six months of treatment, the patient is able to walk without support with less muscle fatigue.

**Fig. 3:**
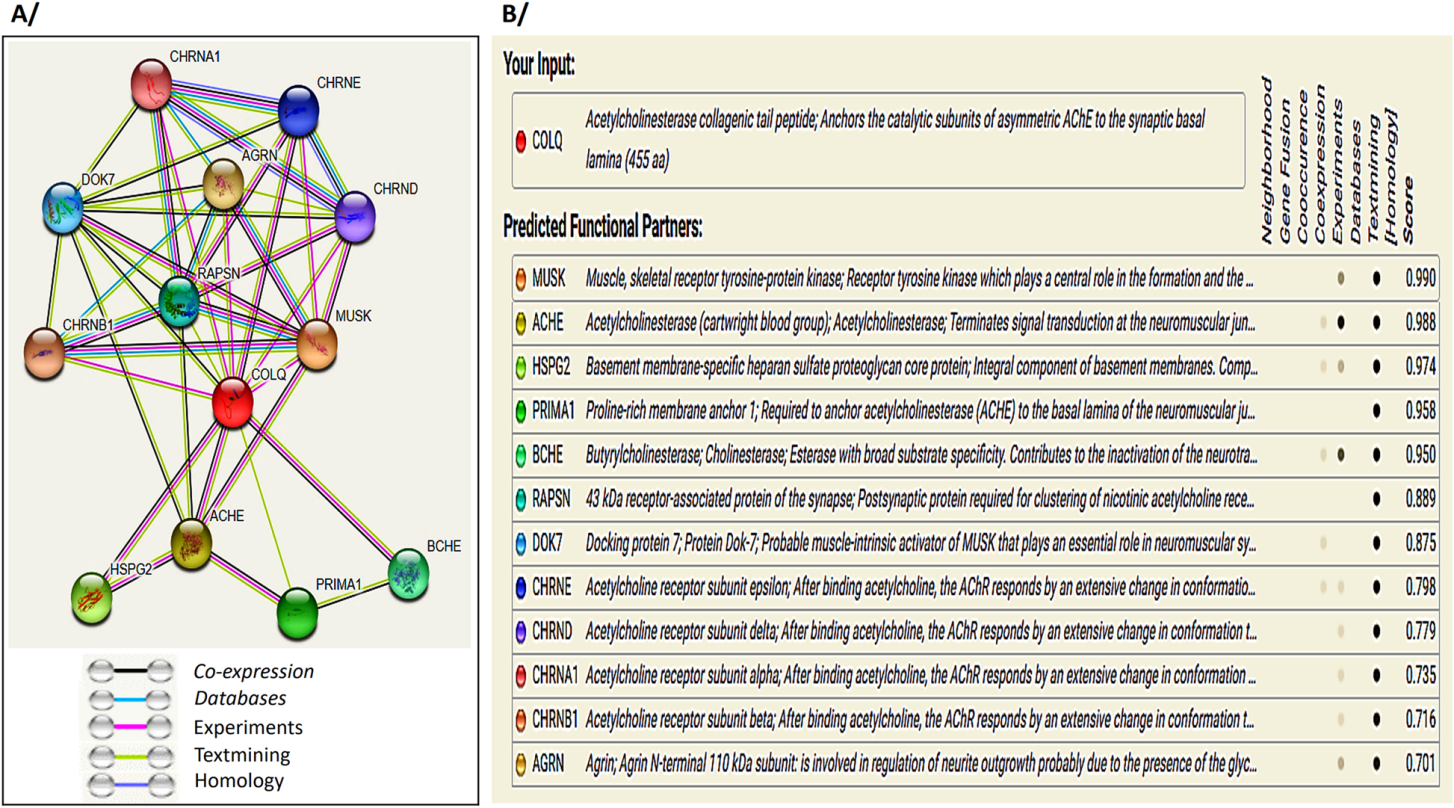
Protein-protein interaction network of COLQ with predicted functional partners. **A** Screenshot of network map of predicted interactions from the STRING database v11.5. **B** Summary view shows predicted protein interactions with the confidence score. The Network nodes represent proteins and edges represent the type of protein-protein associations. Line color indicates the type of interaction evidence between two proteins. Black represents co-expression, turquoise database evidence, pink experimental evidence, light green text mining evidence, and violet protein homology.

To predict the possible COLQ interaction with other proteins, we used STRING database version: 11.5. It is an online service freely available at (https://string-db.org) and it allows the integration of known and predicted associations between proteins including direct (physical) and indirect (functional) interactions. The settings “protein by name” and organism “Homo sapiens” were selected. For “network type”, “required score” and “size cutoff” the options “full STRING network”, “high confidence (0.700)” and “no more than 50 interactors” were chosen, respectively. Each known and predicted association type that contributes to a given network was scored and integrated into a final combined score. The outcome of STRING network for the 50 top protein-coding genes showed that COLQ is interacting with 12 proteins, which grouped according to the process of PPI with a high confidence score that is between 0.701 and 0.990 (Fig. 3). Among these complex network proteins using coexpression (based on RNA expression patterns, and protein co-regulation provided by ProteomeHD-bio.tools), and experiment (detected by x-ray crystallography assay and in vitro assay) evidence as two basic parameters, we observed that AChE protein has interacted to COLQ with the final score 0.988. In addition, *COLQ* mutations affect indirectly AChE with total absence or decrease of its expression level. Therefore, the variability of AChE activity is responsible for the phenotype severity of CMS type-5.

## Discussion and conclusions

Congenital myasthenic syndromes are a heterogeneous group of rare inherited diseases characterized by an abnormal synaptic transmission in the neuromuscular junction, due to pathogenic mutations in genes that encode for proteins involved in the structural organization, maintenance, and function of the motor endplate [12, 13].

According to the last update of GeneReviews, the prevalence of CMS is estimated at one-tenth that of myasthenia gravis (which has a prevalence of 25-125 per one million); however, it may be higher due to limited data, available only from reports of isolated cases [14, 15].

Most likely, the prevalence figures vary from one population to another according to ethnic origin. It may also be underestimated, as CMS may go underdiagnosed if mixed up with one of the many differential diagnoses or if manifesting only with mild clinical features [1, 16].

To date, there are no well-defined clinical and paraclinical diagnostic criteria of CMS but it should be suspected if: (i) early-onset fatigable weakness of skeletal muscles, mainly involving ocular, facial, bulbar, and limb muscles and rarely in late childhood; (ii) family history of congenital hypotonia or previous positive case of a specific subtype of CMS consistent with either autosomal recessive or autosomal dominant inheritance; (iii) decremental EMG response of the compound muscle action potential (CMAP) on low-frequency (2-3 Hz) stimulation or single-fiber EMG studies, compatible with a neuromuscular transmission defect; (iv) an absence of neuromuscular antibodies like anti-acetylcholine receptor (AChR) and anti-MuSK antibodies in the serum; (v) lack of response to immunological treatments and (vi) normal or moderate elevation of serum creatine kinase (CK) concentration [12, 14, 15].

**Fig. 4.**
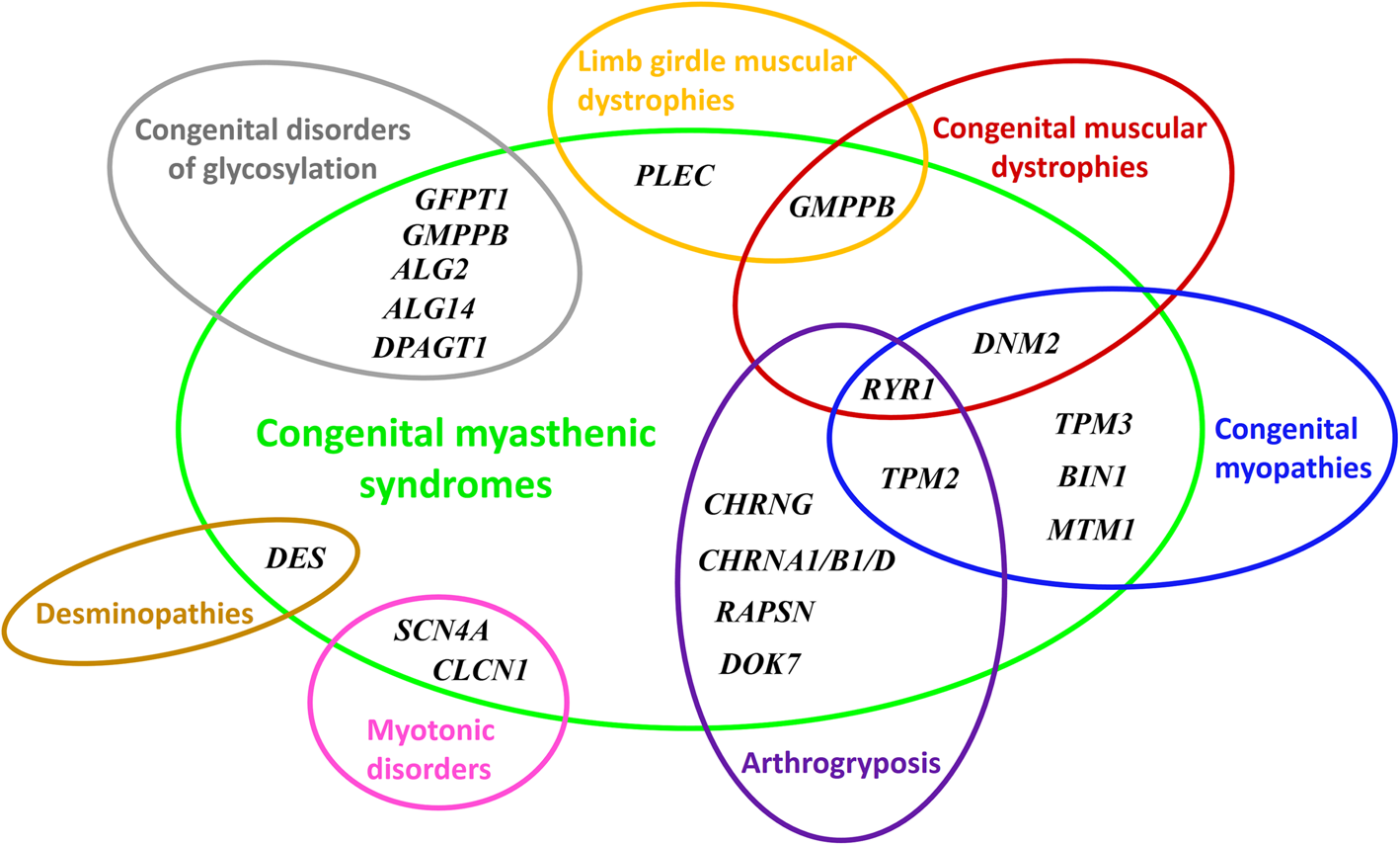
Venn diagram showing possible genes with their different intersections involved in the differential diagnoses between myopathic forms and congenital myasthenic syndromes [2, 17–20]

Interestingly, a long list of differential diagnoses should be taken into consideration before diagnosing CMS patients in early- and late-onset presentations (Fig. 4). Clinicians should be aware of this when facing clinical and paraclinical manifestations, as CMSs share significant overlap with several forms called congenital myasthenic syndrome-like phenotypes [2, 17–19].

Major CMS subtypes are recognized based on molecular genetic studies. There are five most commonly associated CMS genes involved in all CMS cases: *CHRNE* (MIM*100725) mutations account for 30–50% of CMS cases [21, 22], *RAPSN* (MIM*601592) mutations for 15–20% of CMS cases [23]*, COLQ* (MIM*603033) and *DOK7* (MIM*610285) for 10–15% of the CMS cases [3, 24], and *CHAT* (MIM*118490) for 4–5% of CMS cases [15]. All other mutated genes may contribute to less than 1% of CMS cases [15].

The *COLQ* gene encodes a collagen-like strand associated into a triple helix to form a tail that anchors catalytic subunits of acetylcholinesterase (*ACHE*; MIM*100740) to the synaptic basal lamina. Mutations in the *COLQ* gene are uniformly distributed on the three conserved domains of COLQ protein: proline-rich attachment domain (PRAD) in the N-terminal region from exon 1 to exon 4, heparan sulfate proteoglycan-binding domain (HSPBD) in the collagen-like domain from exon 4 to exon 14, including the CNV identified in our patient, and the C-terminal region encodes by genomic exons 15 to 17 [11, 12]. These causative *COLQ* mutations cause an endplate AChE deficiency with an abnormality that varies from normal secretion with decreased activity to the total absence of the protein [25, 26]. They are responsible for a broad range of severity and clinical features (from mild muscle weakness to wheelchair boundness, or early death) for congenital myasthenic syndrome type-5 [27, 28].

To date, there are more than 80 different pathogenic mutations in the *COLQ* gene described in the HGMD database professional release 2021.4 (accessed on 9 May 2022), (http://www.hgmd.cf.ac.uk/ac/gene.php?gene=COLQ). Interestingly, truncating mutations that lead to the production of a transcript with premature termination codon result in premature termination of peptide chain synthesis and the formation of a defective protein, which is subsequently degraded by nonsense-mediated mRNA decay (NMD) in order to prevent the expression of truncated proteins with potentially toxic effects [29].

**Fig. 5.**
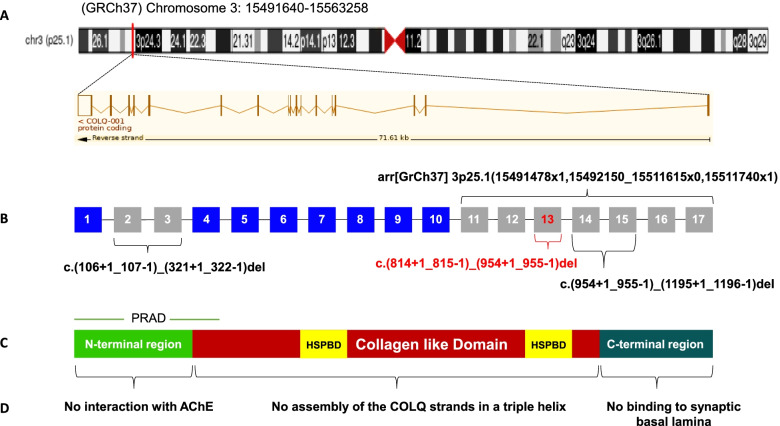
Schematic representation of COLQ domains and the CNV mutations reported in this gene. **A** Genomic location of *COLQ* gene (from UCSC browser GRCh37/hg19: http://genome.ucsc.edu) with the full transcript contains 17 coding exons (from the Ensembl database GRCh37 release 107 - Jul 2022). **B** *COLQ* exons with the three published pathogenic CNVs in the literature and the deletion described in this study (showed in red color). **C** Primary structure of COLQ with its three protein domains. **D** Consequences of mutations in human COLQ protein [11, 32]. Mutations in the N-terminal proline-rich attachment domain (PRAD) prohibit the association of each COLQ strand with an acetylcholinesterase tetramer. Mutations in the central collagen domain containing two heparan sulfate proteoglycan binding (HSPBP) domains cause loss of assembly of the COLQ strands in a triple helix, and mutations in the C-terminal region lead to the synthesis of single- or triple-strands of COLQ-AChE, which are unable to bind to the basal lamina [3, 9, 12, 33].

Copy number variants (CNVs) have been recently defined to be a minimum of 50 bp in size and are currently estimated to explain approximately 10% of all inherited disorders. It highlights the importance of more understanding CNV and its implications for human diseases, particularly in neuromuscular disorders [30, 31]. So far, three large deletions in *COLQ* gene have been previously reported in four unrelated patients before our case (Fig. 5). In 1998, Ohno et al. described a compound heterozygous male patient with early-onset severe myasthenic symptoms carrying a truncation mutation consisting of a large frameshift deletion encompassing exon 2 and exon 3, c.(106+1_107-1)_(321+1_322-1) del inherited from the mother, with a nonsense c.640G>T p.(Glu214*) mutation in exon 11, inherited from the father [9]. In 2016, Wang et al. reported a Chinese girl with a large deletion encompassing exons 14-15 of *COLQ* gene c.(954+1_955-1)_(1195+1_1196-1) del inherited from the mother, and compound heterozygosity with the splicing c.1298+3A>G mutation located at donor (5’) site within intron 16-17 inherited from the father [10]. In 2020, Laforgia et al. identified a homozygous extended deletion encompassing exons 11-17, arr [GrCh37] 3p25.1(15491478x1,15492150_15511615x0,15511740x1) of the *COLQ* gene in a Pakistani male child with severe clinical presentation of CMS [11]. Lastly, in 2021, Luo and their colleagues reported deletion of exons 14-15 at homozygous state in a 12-year-old boy with mild symptoms [29] (Table 1).

**Table 1:** Summary of clinical and molecular data of CMS patients with large deletions in *COLQ* gene reported in literature in addition to our result

Reference		Ohno et ***al***.,1998 [9]	Wang et ***al***.,2016 [10]	Laforgia et ***al***.,2020 [11]	Luo et ***al***.,2021 [29]	Our study
**Sex**		M	F	M	M	F
**Consanguinity**		No	No	Yes	No	Yes
**Age of onset**		Birth	6 years	55 days	18 months	2 months
**Muscle weakness**		Severe	Progressive	Severe	Mild	Mild
**Ocular abnormality**		N/A	No ptosis or double vision	Palpebral ptosis	Ptosis	Ptosis and unilateral partial visual field deficiency
**Respiratory failure**		N/A	N/A	Apnea, respiratory crisis, and cyanosis	**-**	**-**
**Neuromuscular features**		Severe myasthenic symptoms	Weakness of neck muscle, proximal upper and lower limbs	Hypotonia, dystonia, electroclinic fits (chaotic movements, hyperextension of arms and legs)	Incapacity to walk steadily and tended to fall	Facial weakness, axial hypotonia, head drop forward, and weakness of neck muscle
**Deep tendon reflexes**		N/A	N	N/A	N/A	**-**
**CK level**		N/A	N	N/A	N	N
**(anti-AChR)/ (anti-MuSK) antibodies**		(-)/(N/A)	(-)/(-)	(N/A)	(-)/(-)	(-)/(-)
**ENMG**		Decremental response to RNS	Decremental Response to RNS (stimulation at 2 Hz)	Decremental response to RNS (stimulation at 3 Hz) and decrease in the amplitude of MUP	CMAP amplitude increases at low frequency & almost stable at high frequency	Decremental response to RNS (stimulation at 3 Hz)
**Mutation**		c.[(106+1_107-1)_(321+1_322-1)del]; [640G>T]	c.[(954+1_955-1)_(1195+1_1196-1)del]; [1298+3A>G]	arr [GrCh37]3p25.1(15491478x1,15492150_15511615x0,15511740x1)	c.(954+1_955-1)_(1195+1_1196-1)del	c.(814+1_815-1)_(954+1_955-1)del
**Genotype**		Compound heterozygous	Compound heterozygous	Homozygous	Homozygous	Homozygous
**Paternal allele**	**Variation**	c.640G>T p.(Glu214*)	c.1298+3A>G p.(?)	arr [GrCh37]3p25.1(15491478x1,15492150_15511615x0,15511740x1) p.(?)	c.(954+1_955-1)_(1195+1_1196-1) del p.(Ile319Alafs*27)	c.(814+1_815-1)_(954+1_955-1) del p.(Gly272Aspfs*11)
	**Domain**	Distal third of collagen-like domain	C-terminal region	Distal third of collagen-like domain and the entire C-terminal region	Distal third of collagen-like domain and proximal C-terminal region	Distal third of collagen-like domain
**Maternal allele**	**Variation**	c.(106+1_107-1)_(321+1_322-1) del p.(Ala36Glyfs*26)	c.(954+1_955-1)_(1195+1_1196-1) del p.(Ile319Alafs*27)	arr [GrCh37]3p25.1(15491478x1,15492150_15511615x0,15511740x1) p.(?)	c.(954+1_955-1)_(1195+1_1196-1) del p.(Ile319Alafs*27)	c.(814+1_815-1)_(954+1_955-1) del p.(Gly272Aspfs*11)
	**Domain**	N-terminal region (PRAD domain)	Distal third of collagen-like domain and proximal C-terminal region	Distal third of collagen-like domain and the entire C- terminal region	Distal third of collagen-like domain and proximal C-terminal region	Distal third of collagen-like domain
**Treatment**		Response unfavorably to anticholinesterase medications	N/A	Response favorably to 3–4 diaminopyridine and salbutamol	Response favorably to salbutamol	Response favorably to salbutamol
**Evolution**		N/A	Waddling gait, walk until 600 feet at 16 years	Walk with support. NIV during sleep at 20 months	Walk longer at 12 years	Walk without support at 28 months

NCBI accession number: NM_005677.4

*Abbreviations*: − absent; *M* Male, *F* Female, *N/A* Non-available data, *N* Normal, *CK* Creatine kinase, *ENMG* Electroneuromyogram, *RNS* Repetitive nerve stimulation, *MUP* Motor unit potential, *CMAP* Compound muscle action potential, *PRAD* Proline-rich attachment domain, *NIV* Non-invasive ventilation

Recently, the use of biomolecular networks is becoming widespread in modern biology and medicine, not only to clarify the complex biological mechanisms functioning through the understanding of the dynamic interactions occurring in organisms and signaling pathways, but also to highlight the functional association between proteins, and therefore to facilitate the comprehension of disease etiology [34, 35].

Different drugs are now prescribed to treat patients with CMS, with remarkable therapeutic efficiency. Among these CMS treatments, two types of drugs, Ephedrine (sympathomimetic amine), and Salbutamol (selective β2-adrenergic receptor agonist) are recommended as first-line therapy in some CMS types, including *COLQ* CMS [6, 7].

In conclusion, we describe and discuss the clinical and molecular case of a Moroccan patient with a novel copy number variant of the *COLQ* gene. This precise molecular diagnosis allowed us to provide an appropriate course of management to the patient, establish the prognosis, and offer adequate genetic counseling to the family of this rare form of CMS.

## Data Availability

The principal data generated and/or analyzed during the current study are included in the published article. The datasets used in this study are available from the corresponding author on reasonable request.

## Abbreviations

ACHE: Acetylcholinesterase
BAM: Binary alignment map;
CES: Clinical exome sequencing
CK: Creatine kinase
CMAP: Compound muscle action potential
CMS: Congenital myasthenic syndrome
CNV: Copy number variation
COLQ: Collagen-like tail subunit of asymmetric acetylcholinesterase
EMG: Electromyography
HGMD: Human gene mutation database
HSPBD: Heparan sulfate proteoglycan-binding domain
IGV: Integrative genomics viewer
LOF: Loss-of-function
NGS: Next-generation sequencing
PPI: Protein-protein interaction
PRAD: Proline-rich attachment domain
qPCR: Quantitative real-time PCR
